# The Wisconsin Longitudinal Study: New Cognitive, Genetic, Biological, and Social Data and a Diversifying Sample

**DOI:** 10.1093/geroni/igab046.849

**Published:** 2021-12-17

**Authors:** Michal Engelman

## Abstract

The Wisconsin Longitudinal Study (WLS) has followed a sample of one in three Wisconsin high school graduates from the class of 1957 for over 64 years, making it an excellent data source for researchers interested in linking early and midlife characteristics to a wide range of later-life outcomes. The WLS is unique among major studies of aging cohorts for its duration of follow up, the inclusion of siblings, and the combination of rich social and health information. This symposium will provide an overview of the WLS, describe recent data collection and linkages, and introduce ongoing efforts to diversify the educational and racial/ethnic composition of the study sample. WLS data cover nearly every aspect of the participants’ lives from early life socioeconomic background, schooling, family, and work, to physical and mental health, social participation, civic engagement, well-being, and cognition. The study is linked to administrative data including Medicare records, Social Security records, mortality records, and resource data on primary and secondary schools attended by participants as well as characteristics of their employers, industries, and communities of residence. Recent data collection efforts have generated a wealth of new biological and cognitive information, including genetic data collected from saliva and blood samples, measures of the gut microbiome, and derived polygenic scores for educational attainment, cognitive performance, depression, and subjective well-being. The currently-fielding ILIAD effort is implementing rigorous AD diagnostic protocols to track the progression of dementia across cognitive phenotypes. The symposium will conclude with practical information on accessing and using the data.

